# *N*′-(Di-2-pyridylmethyl­ene)benzo­hydrazide

**DOI:** 10.1107/S1600536809022090

**Published:** 2009-06-17

**Authors:** Ismail Warad, Mohammed Al-Nuri, Saud Al-Resayes, Khalid Al-Farhan, Mohamed Ghazzali

**Affiliations:** a Department of Chemistry, King Saud University, PO 2455, Riyadh 11451, Saudi Arabia; b Department of Chemistry, An-Najah National University, PO 7, Nablus, West Bank, Palestinian Territories

## Abstract

In the title Schiff base, C_18_H_14_N_4_O, the amido –NH– unit is connected to one of the two pyridyl N atoms at an N(—H)⋯N distance of 2.624 (2) Å. The mol­ecular packing features an inter­molecular C—H⋯N *R*_2_^2^(6) hydrogen-bonding ring motif.

## Related literature

For medicinal applications of benzohydrazides, see: Raparti *et al.* (2009 ▶); Zhong *et al.* (2007 ▶). For a previous study on the synthesis of benzohyrazide derivatives, see: Abu-El-Halawa *et al.* (2007 ▶). For ring-motif analysis; see: Bernstein *et al.* (1995 ▶); Grell *et al.* (1999 ▶).
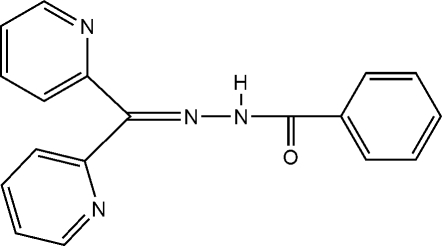


## Experimental

### 

#### Crystal data

C_18_H_14_N_4_O
*M* *_r_* = 302.33
Monoclinic, 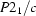

*a* = 8.2741 (5) Å
*b* = 22.1436 (14) Å
*c* = 8.8006 (5) Å
β = 108.974 (2)°
*V* = 1524.82 (15) Å^3^
*Z* = 4
Mo *K*α radiation
μ = 0.09 mm^−1^
*T* = 295 K
0.50 × 0.30 × 0.10 mm

#### Data collection

Rigaku R-AXIS RAPID diffractometer
Absorption correction: multi-scan (*CrystalClear*; Rigaku/MSC, 2007 ▶) *T*_min_ = 0.968, *T*_max_ = 0.989
33115 measured reflections
3491 independent reflections
2329 reflections with *I* > 2δ(*I*)
*R*_int_ = 0.050

#### Refinement

*R*[*F* ^2^ > 2σ(*F* ^2^)] = 0.053
*wR*(*F* ^2^) = 0.169
*S* = 0.99
3491 reflections
208 parameters
H-atom parameters constrained
Δρ_max_ = 0.15 e Å^−3^
Δρ_min_ = −0.24 e Å^−3^

### 

Data collection: *CrystalClear* (Rigaku/MSC, 2007 ▶); cell refinement: *CrystalClear*; data reduction: *CrystalClear*; program(s) used to solve structure: *SHELXS97* (Sheldrick, 2008 ▶); program(s) used to refine structure: *SHELXL97* (Sheldrick, 2008 ▶); molecular graphics: *DIAMOND* (Brandenburg, 2006 ▶) and *PLUTO* (Motherwell *et al.*, 1999 ▶); software used to prepare material for publication: *publCIF* (Westrip, 2009 ▶).

## Supplementary Material

Crystal structure: contains datablocks I, global. DOI: 10.1107/S1600536809022090/ng2595sup1.cif

Structure factors: contains datablocks I. DOI: 10.1107/S1600536809022090/ng2595Isup2.hkl

Additional supplementary materials:  crystallographic information; 3D view; checkCIF report

## Figures and Tables

**Table 1:** Hydrogen-bond geometry (Å, °)

*D*—H⋯*A*	*D*—H	H⋯*A*	*D*⋯*A*	*D*—H⋯*A*
N1—H7⋯N3	0.86	1.94	2.624 (2)	136
C9—H9⋯N4	0.93	2.45	2.973 (2)	115

**Fig. 1.**
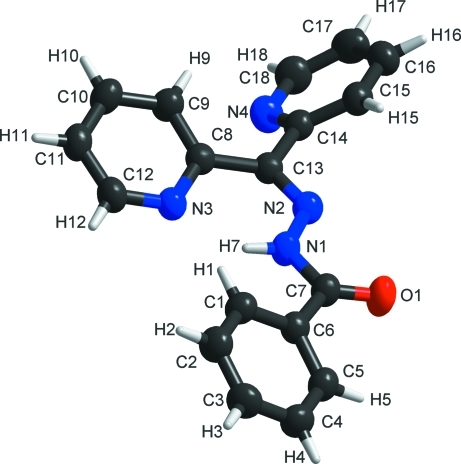
Perspective drawings of the title compound showing the atom-numbering scheme. The atomic displacement ellipsoids are shown at the 50% probability level. Hydrogen atoms are drawn as spheres of arbitrary radius.

**Fig. 2.**
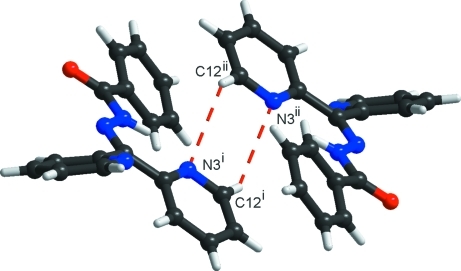
C— H···N intermolecular hydrogen bonding pattern of the title compound with hydrogen bonding shown as broken lines. For symmetry codes; i: 1 + x, y, z and ii: 1 - x, 1 - y, 2 - z.

## supplementary crystallographic information

### Comment

Benzohydrazide derivatives were known as good antitumor and antimycobacterial agent (Raparti *et al.* 2009; Zhong *et al.* 2007). The molecular packing of the title compound is supported by C— H···N intermolecular hydrogen bondings at D···A distance of 2.956 (1) and D— H···A angle of 128.92 (1)° that is recognized by *R*_2_^2^(6) second order ring motif, as earlier defined (Bernstein *et al.* 1995; Grell *et al.* 1999) and calculated with Pluto (Motherwell *et al.*, 1999), see Figure 2.

### Experimental

Equimolar amounts of di-2-pyridyl ketone and benzohydrazide were mixed in ethanol.(Abu-El-Halawa *et al.* 2007) Five drops of conc. HCl were added and the mixture was refluxed for 8–10 h. After cooling, distilled water was added up to 1:3 volume ratio followed by addition of several drops of sodium hydroxide solution. The product was re-crystallized twice by water. IR, cm^-1^ (CHCl_3_): 3280, 3210, 3100, 3020, 1660, 1620, 1600, 1580, 1480, 1450, 1350, 1160, 1040, 770, 660. ^1^H NMR (p.p.m.): 10.70 (bs, 1H exchangeable with D_2_O), 8.83 (cp, 2H of two pyridine rings), 8.02 (cp, 2H of two pyridine rings), 7.99 (cp, 2H of two pyridine rings), 7.95 (cp, 2H of benzene ring), 7.62 (cp, 2H of two pyridine rings), 7.44 (cp, 2H of benzene ring) p.p.m.; ^13^C NMR (p.p.m.): 163.0, 155.6, 152.6, 149.2, 136.1, 134.2, 132.2, 128.9, 127.5, 126.2, 123.9.

### Refinement

Hydrogen atoms were refined isotropically and were constrained to the ideal geometry using an appropriate riding model with *U*_iso_(H) fixed at 1.2 times U_eq _of the pivot atom.

### Crystal data

**Table:**

C_18_H_14_N_4_O	*F*(000) = 632
*M**_r_* = 302.33	*D*_x_ = 1.317 Mg m^−^^3^
Monoclinic, *P*2_1_/*c*	Mo *K*α radiation, λ = 0.71075 Å
Hall symbol: -P 2ybc	Cell parameters from 880 reflections
*a* = 8.2741 (5) Å	θ = 3.0–27.4°
*b* = 22.1436 (14) Å	µ = 0.09 mm^−^^1^
*c* = 8.8006 (5) Å	*T* = 295 K
β = 108.974 (2)°	Block, colourless
*V* = 1524.82 (15) Å^3^	0.50 × 0.30 × 0.10 mm
*Z* = 4	

### Data collection

**Table:**

Rigaku R-AXIS RAPID diffractometer	3491 independent reflections
Radiation source: fine-focus sealed tube	2329 reflections with *I* > 2δ(*I*)
graphite	*R*_int_ = 0.050
ω scans	θ_max_ = 27.5°, θ_min_ = 3.1°
Absorption correction: multi-scan (*CrystalClear*; Rigaku/MSC, 2007)	*h* = −10→10
*T*_min_ = 0.968, *T*_max_ = 0.989	*k* = −28→28
33115 measured reflections	*l* = −11→11

### Refinement

**Table:**

Refinement on *F*^2^	Primary atom site location: structure-invariant direct methods
Least-squares matrix: full	Secondary atom site location: difference Fourier map
*R*[*F*^2^ > 2σ(*F*^2^)] = 0.053	Hydrogen site location: inferred from neighbouring sites
*wR*(*F*^2^) = 0.169	H-atom parameters constrained
*S* = 0.99	*w* = 1/[σ^2^(*F*_o_^2^) + (0.1092*P*)^2^ + 0.076*P*] where *P* = (*F*_o_^2^ + 2*F*_c_^2^)/3
3491 reflections	(Δ/σ)_max_ < 0.001
208 parameters	Δρ_max_ = 0.15 e Å^−^^3^
0 restraints	Δρ_min_ = −0.24 e Å^−^^3^

### Special details

**Table:**

Geometry. All e.s.d.'s (except the e.s.d. in the dihedral angle between two l.s. planes) are estimated using the full covariance matrix. The cell e.s.d.'s are taken into account individually in the estimation of e.s.d.'s in distances, angles and torsion angles; correlations between e.s.d.'s in cell parameters are only used when they are defined by crystal symmetry. An approximate (isotropic) treatment of cell e.s.d.'s is used for estimating e.s.d.'s involving l.s. planes.
Refinement. Refinement of *F*^2^ against ALL reflections. The weighted *R*-factor *wR* and goodness of fit *S* are based on *F*^2^, conventional *R*-factors *R* are based on *F*, with *F* set to zero for negative *F*^2^. The threshold expression of *F*^2^ > σ(*F*^2^) is used only for calculating *R*-factors(gt) *etc*. and is not relevant to the choice of reflections for refinement. *R*-factors based on *F*^2^ are statistically about twice as large as those based on *F*, and *R*- factors based on ALL data will be even larger.

### Fractional atomic coordinates and isotropic or equivalent isotropic displacement parameters (Å^2^)

**Table:**

	*x*	*y*	*z*	*U*_iso_*/*U*_eq_	
O1	0.17708 (18)	0.55123 (6)	0.48480 (14)	0.0649 (4)	
N1	0.21198 (18)	0.51374 (6)	0.73562 (16)	0.0485 (4)	
H7	0.1902	0.5186	0.8240	0.058*	
C1	0.1073 (2)	0.62760 (7)	0.8228 (2)	0.0513 (4)	
H1	0.1787	0.6045	0.9058	0.062*	
N2	0.29967 (18)	0.46379 (6)	0.71474 (16)	0.0472 (3)	
C2	0.0312 (2)	0.67909 (8)	0.8581 (2)	0.0598 (5)	
H2	0.0526	0.6907	0.9644	0.072*	
N3	0.22378 (18)	0.47159 (6)	1.01763 (16)	0.0509 (4)	
C3	−0.0768 (2)	0.71315 (8)	0.7347 (2)	0.0604 (5)	
H3	−0.1280	0.7477	0.7583	0.072*	
N4	0.4144 (2)	0.31839 (6)	0.84066 (19)	0.0550 (4)	
C4	−0.1087 (2)	0.69618 (8)	0.5779 (2)	0.0590 (5)	
H4	−0.1829	0.7189	0.4956	0.071*	
C5	−0.0308 (2)	0.64538 (8)	0.5414 (2)	0.0524 (4)	
H5	−0.0509	0.6346	0.4346	0.063*	
C6	0.0777 (2)	0.61027 (7)	0.66396 (19)	0.0455 (4)	
C7	0.1590 (2)	0.55591 (7)	0.61664 (19)	0.0485 (4)	
C8	0.3333 (2)	0.42967 (7)	0.99547 (18)	0.0432 (4)	
C9	0.4310 (2)	0.39466 (8)	1.1233 (2)	0.0527 (4)	
H9	0.5048	0.3655	1.1072	0.063*	
C10	0.4182 (3)	0.40325 (8)	1.2745 (2)	0.0572 (5)	
H10	0.4845	0.3804	1.3609	0.069*	
C11	0.3068 (2)	0.44583 (8)	1.2965 (2)	0.0533 (4)	
H11	0.2958	0.4523	1.3971	0.064*	
C12	0.2125 (2)	0.47854 (8)	1.1649 (2)	0.0551 (4)	
H12	0.1363	0.5072	1.1789	0.066*	
C13	0.3515 (2)	0.42534 (7)	0.83260 (18)	0.0433 (4)	
C14	0.4492 (2)	0.37362 (7)	0.79543 (18)	0.0444 (4)	
C15	0.5690 (2)	0.38299 (8)	0.71803 (19)	0.0514 (4)	
H15	0.5880	0.4216	0.6856	0.062*	
C16	0.6594 (2)	0.33445 (9)	0.6899 (2)	0.0611 (5)	
H16	0.7415	0.3399	0.6399	0.073*	
C17	0.6260 (3)	0.27757 (9)	0.7371 (2)	0.0645 (5)	
H17	0.6853	0.2440	0.7199	0.077*	
C18	0.5033 (3)	0.27192 (8)	0.8099 (2)	0.0617 (5)	
H18	0.4802	0.2334	0.8399	0.074*	

### Atomic displacement parameters (Å^2^)

**Table:**

	*U*^11^	*U*^22^	*U*^33^	*U*^12^	*U*^13^	*U*^23^
O1	0.0939 (10)	0.0613 (8)	0.0480 (7)	0.0160 (7)	0.0345 (7)	0.0053 (6)
N1	0.0645 (9)	0.0402 (7)	0.0455 (8)	0.0074 (6)	0.0244 (7)	0.0017 (5)
C1	0.0561 (10)	0.0503 (9)	0.0458 (9)	0.0045 (7)	0.0141 (8)	0.0005 (7)
N2	0.0561 (8)	0.0404 (7)	0.0474 (8)	0.0030 (6)	0.0197 (6)	−0.0015 (6)
C2	0.0679 (12)	0.0604 (11)	0.0519 (10)	0.0054 (9)	0.0209 (9)	−0.0086 (8)
N3	0.0565 (9)	0.0531 (8)	0.0476 (8)	0.0065 (6)	0.0230 (6)	0.0016 (6)
C3	0.0657 (12)	0.0517 (10)	0.0663 (12)	0.0110 (8)	0.0251 (10)	−0.0015 (8)
N4	0.0674 (10)	0.0388 (7)	0.0649 (9)	−0.0004 (6)	0.0300 (8)	−0.0024 (6)
C4	0.0631 (11)	0.0563 (10)	0.0579 (11)	0.0115 (8)	0.0204 (9)	0.0128 (8)
C5	0.0606 (10)	0.0517 (9)	0.0463 (9)	0.0027 (8)	0.0192 (8)	0.0053 (7)
C6	0.0506 (9)	0.0430 (8)	0.0454 (9)	−0.0013 (7)	0.0192 (7)	0.0018 (6)
C7	0.0570 (10)	0.0464 (9)	0.0444 (9)	0.0018 (7)	0.0197 (7)	0.0023 (7)
C8	0.0476 (9)	0.0383 (8)	0.0456 (9)	−0.0028 (6)	0.0176 (7)	−0.0025 (6)
C9	0.0607 (10)	0.0493 (9)	0.0488 (9)	0.0084 (8)	0.0188 (8)	0.0000 (7)
C10	0.0720 (12)	0.0539 (10)	0.0439 (9)	0.0034 (8)	0.0165 (8)	0.0025 (7)
C11	0.0668 (11)	0.0527 (10)	0.0452 (9)	−0.0066 (8)	0.0249 (8)	−0.0041 (7)
C12	0.0623 (11)	0.0565 (10)	0.0538 (10)	0.0040 (8)	0.0289 (9)	−0.0021 (8)
C13	0.0477 (9)	0.0407 (8)	0.0427 (8)	−0.0021 (6)	0.0165 (7)	−0.0027 (6)
C14	0.0495 (9)	0.0417 (8)	0.0409 (8)	−0.0002 (6)	0.0132 (7)	−0.0025 (6)
C15	0.0564 (10)	0.0511 (9)	0.0487 (9)	−0.0005 (7)	0.0197 (8)	0.0007 (7)
C16	0.0597 (11)	0.0703 (12)	0.0584 (11)	0.0085 (9)	0.0264 (9)	−0.0030 (9)
C17	0.0699 (12)	0.0594 (11)	0.0622 (12)	0.0174 (9)	0.0189 (10)	−0.0082 (9)
C18	0.0770 (13)	0.0423 (9)	0.0672 (12)	0.0041 (8)	0.0255 (10)	−0.0031 (8)

### Geometric parameters (Å, °)

**Table:**

O1—C7	1.2217 (18)	C6—C7	1.502 (2)
N1—C7	1.365 (2)	C8—C9	1.389 (2)
N1—N2	1.3677 (17)	C8—C13	1.492 (2)
N1—H7	0.8600	C9—C10	1.382 (2)
C1—C2	1.386 (2)	C9—H9	0.9300
C1—C6	1.392 (2)	C10—C11	1.375 (2)
C1—H1	0.9300	C10—H10	0.9300
N2—C13	1.302 (2)	C11—C12	1.374 (2)
C2—C3	1.384 (3)	C11—H11	0.9300
C2—H2	0.9300	C12—H12	0.9300
N3—C12	1.338 (2)	C13—C14	1.498 (2)
N3—C8	1.355 (2)	C14—C15	1.389 (2)
C3—C4	1.371 (3)	C15—C16	1.377 (2)
C3—H3	0.9300	C15—H15	0.9300
N4—C18	1.343 (2)	C16—C17	1.382 (3)
N4—C14	1.3458 (19)	C16—H16	0.9300
C4—C5	1.385 (2)	C17—C18	1.371 (3)
C4—H4	0.9300	C17—H17	0.9300
C5—C6	1.394 (2)	C18—H18	0.9300
C5—H5	0.9300		
			
C7—N1—N2	120.17 (13)	C10—C9—C8	119.78 (15)
C7—N1—H7	119.9	C10—C9—H9	120.1
N2—N1—H7	119.9	C8—C9—H9	120.1
C2—C1—C6	120.52 (16)	C11—C10—C9	119.62 (16)
C2—C1—H1	119.7	C11—C10—H10	120.2
C6—C1—H1	119.7	C9—C10—H10	120.2
C13—N2—N1	118.27 (12)	C12—C11—C10	117.75 (15)
C3—C2—C1	119.78 (16)	C12—C11—H11	121.1
C3—C2—H2	120.1	C10—C11—H11	121.1
C1—C2—H2	120.1	N3—C12—C11	123.82 (16)
C12—N3—C8	118.61 (14)	N3—C12—H12	118.1
C4—C3—C2	120.29 (16)	C11—C12—H12	118.1
C4—C3—H3	119.9	N2—C13—C8	127.81 (14)
C2—C3—H3	119.9	N2—C13—C14	112.79 (13)
C18—N4—C14	116.92 (15)	C8—C13—C14	119.29 (13)
C3—C4—C5	120.30 (16)	N4—C14—C15	122.42 (15)
C3—C4—H4	119.8	N4—C14—C13	116.60 (14)
C5—C4—H4	119.8	C15—C14—C13	120.98 (14)
C4—C5—C6	120.28 (16)	C16—C15—C14	119.18 (16)
C4—C5—H5	119.9	C16—C15—H15	120.4
C6—C5—H5	119.9	C14—C15—H15	120.4
C1—C6—C5	118.80 (15)	C15—C16—C17	118.97 (17)
C1—C6—C7	123.43 (14)	C15—C16—H16	120.5
C5—C6—C7	117.76 (14)	C17—C16—H16	120.5
O1—C7—N1	124.24 (15)	C18—C17—C16	118.30 (17)
O1—C7—C6	122.38 (14)	C18—C17—H17	120.8
N1—C7—C6	113.37 (13)	C16—C17—H17	120.9
N3—C8—C9	120.41 (14)	N4—C18—C17	124.18 (17)
N3—C8—C13	117.62 (13)	N4—C18—H18	117.9
C9—C8—C13	121.88 (14)	C17—C18—H18	117.9

### Hydrogen-bond geometry (Å, °)

**Table:**

*D*—H···*A*	*D*—H	H···*A*	*D*···*A*	*D*—H···*A*
N1—H7···N3	0.86	1.94	2.624 (2)	136
C9—H9···N4	0.93	2.45	2.973 (2)	115
